# The phenotypic expression of mitochondrial tRNA-mutations can be modulated by either mitochondrial leucyl-tRNA synthetase or the C-terminal domain thereof

**DOI:** 10.3389/fgene.2015.00113

**Published:** 2015-03-23

**Authors:** Carla Giordano, Veronica Morea, Elena Perli, Giulia d’Amati

**Affiliations:** ^1^Department of Radiological, Oncological and Pathological Sciences, Sapienza University of RomeRome, Italy; ^2^National Research Council of Italy, Institute of Molecular Biology and Pathology, Department of Biochemical Sciences, Sapienza University of RomeRome, Italy; ^3^Pasteur Institute-Cenci Bolognetti FoundationRome, Italy

**Keywords:** mitochondria, mt-tRNA, aminoacyl-tRNA synthetase, mitochondrial disease, molecular therapy

## Abstract

Mutations in mitochondrial (mt) DNA determine important human diseases. The majority of the known pathogenic mutations are located in transfer RNA (tRNA) genes and are responsible for a wide range of currently untreatable disorders. Experimental evidence both in yeast and in human cells has shown that the detrimental effects of mt-tRNA point mutations can be attenuated by increasing the expression of the cognate mt-aminoacyl-tRNA synthetases (aaRSs). In addition, constitutive high levels of isoleucyl-tRNA syntethase have been shown to reduce the penetrance of a homoplasmic mutation in mt-tRNA^Ile^ in a small kindred. More recently, we showed that the isolated carboxy-terminal domain of human mt-leucyl tRNA synthetase (LeuRS-Cterm) localizes to mitochondria and ameliorates the energetic defect in *trans*mitochondrial cybrids carrying mutations either in the cognate mt-tRNA^Leu(UUR)^ or in the non-cognate mt-tRNA^Ile^ gene. Since the mt-LeuRS-Cterm does not possess catalytic activity, its rescuing ability is most likely mediated by a chaperon-like effect, consisting in the stabilization of the tRNA structure altered by the mutation. All together, these observations open potential therapeutic options for mt-tRNA mutations-associated diseases.

## Introduction

Mutations in genes coding for mt-tRNAs (*MTT*s) are responsible for a wide range of currently untreatable pathologies. Clinical presentation may occur at any age, ranging from isolated organ-specific disorders such as cardiomyopathy or hearing loss, to multisystem diseases including myopathies, encephalopathies, deafness, diabetes and others (Yarham et al., 2010). Most mt-tRNA pathogenic mutations are heteroplasmic (i.e., mutant and wild type molecules co-exist within the same cell), and manifest clinically only when mutated mtDNA exceeds a threshold level, typically 60–90% (Greaves et al., 2012). However, homoplasmic pathogenic mutations (a condition where all mtDNA molecules are mutated in the cell) have been reported, either in association with tissue-specific disorders (i.e., maternally inherited cardiomyopathy; Taylor et al., 2003; Perli et al., 2012) or with devastating multisystem diseases (McFarland et al., 2002; Limongelli et al., 2004).

Mitochondrial (mt) tRNA mutations would be expected to cause impaired mt protein synthesis (i.e., defective translation of the 13 mtDNA-encoded protein subunits of the respiratory chain), leading to a generalized OXPHOS defect. The mechanism by which mutations induce a quantitative and/or qualitative defect of mt translation is complex and not completely understood. Biochemical characterizations of mutant tRNAs transcribed *in vitro* and studies on patients derived *trans*mitochondrial cybrids (herein cybrids) have shown that mutations may negatively affect different steps of tRNA biogenesis and/or functioning, including processing, post-transcriptional modification, aminoacylation and translation (see for Review Yarham et al., 2010; Suzuki et al., 2011). Most pathogenic mutations directly affect mt-tRNA tertiary structure and stability. This, in turn, can hamper mt-tRNA interactions required for productive protein synthesis e.g., interactions with: (1) enzymes that perform post-transcriptional modifications essential for translational accuracy and efficiency; (2) cognate aaRSs or other tRNA synthetases, which may lead to non-charged or mischarged tRNA formation; or (3) translation factors or ribosome, which would affect the rate or accuracy of translational initiation or elongation. These alterations would be expected to cause generalized translation defects and, therefore, decreased levels of mtDNA-encoded polypeptides. As mentioned above, mutations may also affect tRNA recognition by enzymes not directly involved in translation, e.g., enzymes that process mtDNA polycistronic transcripts, thus leading to decreased steady-state levels of mature mt-tRNA available for aminoacylation.

The most frequent and extensively studied mt-tRNA mutation is m.3243A>G, one of the 32 disease-associated mutations within the *MTTL1* gene coding for mt-tRNA^Leu(UUR)^ (http://www.mitomap.org/bin/view.pl/MITOMAP/MutationsRNA). The effects of this mutation are reported in **Table 1**.

**Table 1:** Reported effects of m.3243A>G mutation on tRNA^**Leu(UUR)**^ structure, processing and function.

Effects of m.3243A>G mutation on tRNA^Leu(UUR)^ structure, processing and function	References
Disruption of the L-shaped tertiary structure and decreased stability of the mutant tRNA	Wittenhagen and Kelley (2002), Sohm et al. (2003)
Dimerization of mutated tRNAs	Wittenhagen and Kelley (2002), Roy et al. (2005)
Reduced tRNA^Leu(UUR)^ steady-state levels	Park et al. (2003)
Reduction of 3’-end processing efficiency	Koga et al. (1993), Levinger et al. (2004)
Accumulation of processing intermediates (RNA19S)	King et al. (1992)
Defect of uridine modification at the anticodon wobble position	Yasukawa et al. (2000), Kirino et al. (2005)
Decreased aminoacylation level and efficiency	Borner et al. (2000), Chomyn et al. (2000), Park et al. (2003), Sohm et al. (2003).

An important issue that remains to be elucidated is the remarkable heterogeneity of clinical phenotypes. the heteroplasmic m.3243A>G mutation has been reported to occur in association with a number of clinical syndromes such as encephalomyopathy, lactic acidosis and stroke-like episodes (MELAS) syndrome; chronic progressive external ophthalmoplegia (CPEO); and maternally inherited diabetes and deafness (MIDD). Both the specific energetic needs of affected tissues and, in case of heteroplasmic mutations, the variable ratios of wild-type and mutant tRNAs in different tissues, contribute to generating phenotypic variability. However, other factors, such as the effect of environment, the mt genetic background, and the interaction with nuclear genes involved in different steps of mt-tRNA processing and modification, may also affect the phenotypic expression of the mutations. This is exemplified by the case of mt-tRNA homoplasmic mutations which show an extremely variable clinical penetrance even within the same family, despite in all individuals all mtDNA molecules are mutated.

## The Penetrance of mt-tRNAs Mutations can be Modulated by Over-Expression of mt-aaRS

Proteins interacting with mt-tRNAs are able to effectively rescue the pathological phenotypes due to point mutations in mt-tRNA genes. This has been first demonstrated by the over-expression of the nuclear gene coding for mt EF-Tu in yeast *Saccharomyces cerevisiae* strains carrying point mutations in *MTTL1* gene, equivalent to those associated with human diseases (Feuermann et al., 2003). EF-Tu is an evolutionarily conserved elongation factor, which plays a central role in the translation process by binding the aminoacylated tRNA, protecting it from hydrolysis, and carrying it to the ribosome. The results obtained in the yeast model have been paralleled in human cell lines. Overexpression of mt elongation factors EF-Tu and EFG2 partially rescues the severe respiratory chain deficiency of myoblasts carrying the MELAS-associated m.3243A>G mutation in *MTTL1* at homoplasmic levels (Sasarman et al., 2008). Subsequently, the detrimental effects of mt-tRNA point mutations have been shown to be modulated by the expression levels of additional genes, in particular mt aaRSs. Studies on the yeast model revealed that over-expression of the nuclear genes *NAM2* and *HTS1*, coding respectively for yeast mt-LeuRS and mt-HisRS (Natsoulis et al., 1986; Zagorski et al., 1991) rescues the growth-defective phenotype of yeast strains carrying human equivalent point mutations in the cognate mt-tRNAs (De Luca et al., 2006). Likewise, over-expression of mt-LeuRS has been shown to correct the respiratory chain deficiency of human patients-derived cybrids harboring the m.3243A>G mutation in the *MTTL1* gene. (Park et al., 2008; Li and Guan, 2010).

The ability to modulate the effects of pathogenic mt-tRNA mutations in human cells has been shown to be shared by other mt-aaRSs belonging, like LeuRS, to Class I and subclass a. As an example, the steady state levels of mutated mt-tRNA^Val^ were partially restored by over-expressing the cognate mt valyl-tRNA synthetase (ValRS) in cybrid cell lines (Rorbach et al., 2008) More recently, our group has shown that constitutively high levels of mt-IleRS are associated with reduced penetrance of the homoplasmic m.4277T>C mt-tRNA^Ile^ mutation, which causes hypertrophic cardiomyopathy. Our *in vivo* findings were paralleled by results in mutant cybrids obtained by over-expression of mt-IleRS (Perli et al., 2012).

Aminoacyl-tRNA synthetases are ubiquitously expressed enzymes that catalyze the specific attachment of each of the 20 amino acids with cognate tRNAs bearing the correct anticodon triplet. Aminoacylation is a two-step reaction in which amino acids are first activated by ATP, forming an intermediate aminoacyl-adenylate, and then transferred to the 3′-end of tRNA to form the aminoacyl-tRNA end-product (Ibba and Soll, 2000). Human cells contain aaRSs specific to cytoplasm, mitochondria or, in some cases, both, depending on the cellular compartment where they exert their catalytic activity and the set of tRNAs used as substrates. Based on the architecture of their catalytic binding domain, aaRSs are grouped in two classes, I and II (Schimmel, 1987; Cusack et al., 1990; Eriani et al., 1990). Class I aaRSs are specific for amino acids Val, Leu, Ile, Met, Cys, Glu, Gln, Tyr, Trp, and Arg. Their active site is located in a Rossman fold nucleotide-binding catalytic domain (made of six parallel β-strands alternating to α-helices; Li et al., 1992). Class II aaRSs are specific for amino acids Gly, Ala, Ser, Thr, Asn, Asp, Lys, His, Phe, and Pro. They are mostly dimeric or multimeric, their active site is contained in an anti-parallel β-sheet with flanking α-helices, and they share at least three conserved regions (Cusack et al., 1991; Schimmel, 1991; Perona et al., 1993). Both class I and class II aaRSs are further divided into a, b and c subclasses, each comprising enzymes sharing sequence, structure and function similarities. All aaRSs contain both a catalytic and an anticodon recognition domain, which are required to catalyze the aminoacylation reaction and recognize the tRNA molecule specific for each cognate amino acid, respectively. To ensure translational fidelity, several aaRSs contain an additional editing domain able to deacylate mischarged amino acids, with the aim of preventing insertion of incorrect amino acids during protein synthesis (Beebe et al., 2008; Schimmel, 2008; Martinis and Boniecki, 2010; Yao and Fox, 2013).

Although the main aaRSs function consists in charging tRNAs with their cognate amino acids in the initiation step of protein synthesis, a number of additional functions have been recently discovered to be carried out by these enzymes. During evolution, cytoplasmic aaRSs have acquired additional non-catalytic domains and insertions, dispensable for aminoacylation, which are involved in pathways of apoptosis, angiogenesis, immune response, tumorigenesis and inflammation (Delarue and Moras, 1993; Guo and Schimmel, 2013; Lo et al., 2014). Initially, analogous domains with non-catalytic functions had not been identified in mammalian mt aaRSs. However, domains of both mt-TyrRS from *Neurospora crassa* and mt-LeuRS from *S. cerevisiae* have been shown to be essential factors for the splicing of several mt RNA group I introns (Akins and Lambowitz, 1987; Hsu et al., 2006). Deletion analysis showed that the splicing function of yeast mt LeuRS resided in a sixty-amino acid region at the carboxy-terminal end of the enzyme and that deletion of this region had no impact on the aminoacylation activity (Li et al., 1996). Interestingly, the homologous human mt-LeuRS-Cterm conserves the splicing activity although there is no requirement for intron splicing following human mtDNA expression (Houman et al., 2000).

## The Carboxy-Terminal Domain of mt-LeuRS is able to Rescue Defects Associated with both Cognate and Non-Cognate mt-tRNA Mutations in Human Cells

An important feature of the yeast mt-LeuRS-Cterm, in view of potential therapeutic developments, is the ability to rescue defective phenotypes associated with human-equivalent point mutations in yeast mt-tRNAs (Francisci et al., 2011). Recently, we and others have shown that human mt-LeuRS-Cterm: (i) is the region necessary and sufficient to ameliorate the mt defects of patient-derived cybrids carrying mutations in both cognate and non-cognate mt-tRNAs (namely, mt-tRNA^Leu(UUR)^, mt-tRNA^Ile^, mt-tRNA^Val^, all of which are aminoacylated by Class Ia aaRSs); and (ii) has a higher rescuing activity than the whole mt-LeuRS toward all of the tested mutations (Hornig-Do et al., 2014; Perli et al., 2014).

The demonstration that the catalytic function of mt-LeuRS-Cterm is not required for its rescuing activity and the interactions occurring between the LeuRS-Cterm domain and the cognate tRNA in experimentally determined three-dimensional structures (see below) led us to speculate that the ability of this domain to correct the biochemical phenotype associated with pathogenic mt-tRNA mutations may be ascribed to a ‘chaperone-like’ effect. Both human (Perli et al., 2012) and yeast (Francisci et al., 2005) mt-tRNAs bearing point mutations that determine a defective phenotype have been previously shown to undergo conformational and/or aminoacylation defects. We hypothesize that, by directly interacting with the mutated mt-tRNA, the mt-LeuRS-Cterm stabilizes a native-like tRNA conformation which would be, in turn, better equipped at establishing interactions with proteins and other macromolecular partners required for protein synthesis, and/or more resistant toward degradation events.

## Structural Basis of the Ability of mt-LeuRS-Cterm to Interact with Cognate and Non-Cognate mt-tRNAs

The hypothesis that the rescuing activity of human mt-LeuRS-Cterm is mediated by a direct interaction with mutated tRNA molecules is supported by the results of our *in vitro* surface plasmon resonance experiments. These demonstrated that mt-LeuRS-Cterm is able to directly and specifically interact with human cognate mt-tRNA^Leu(UUR)^ with high affinity and stability, and with non-cognate mt-tRNA^Ile^ with 4-fold lower affinity (Perli et al., 2014).

**FIGURE 1:**
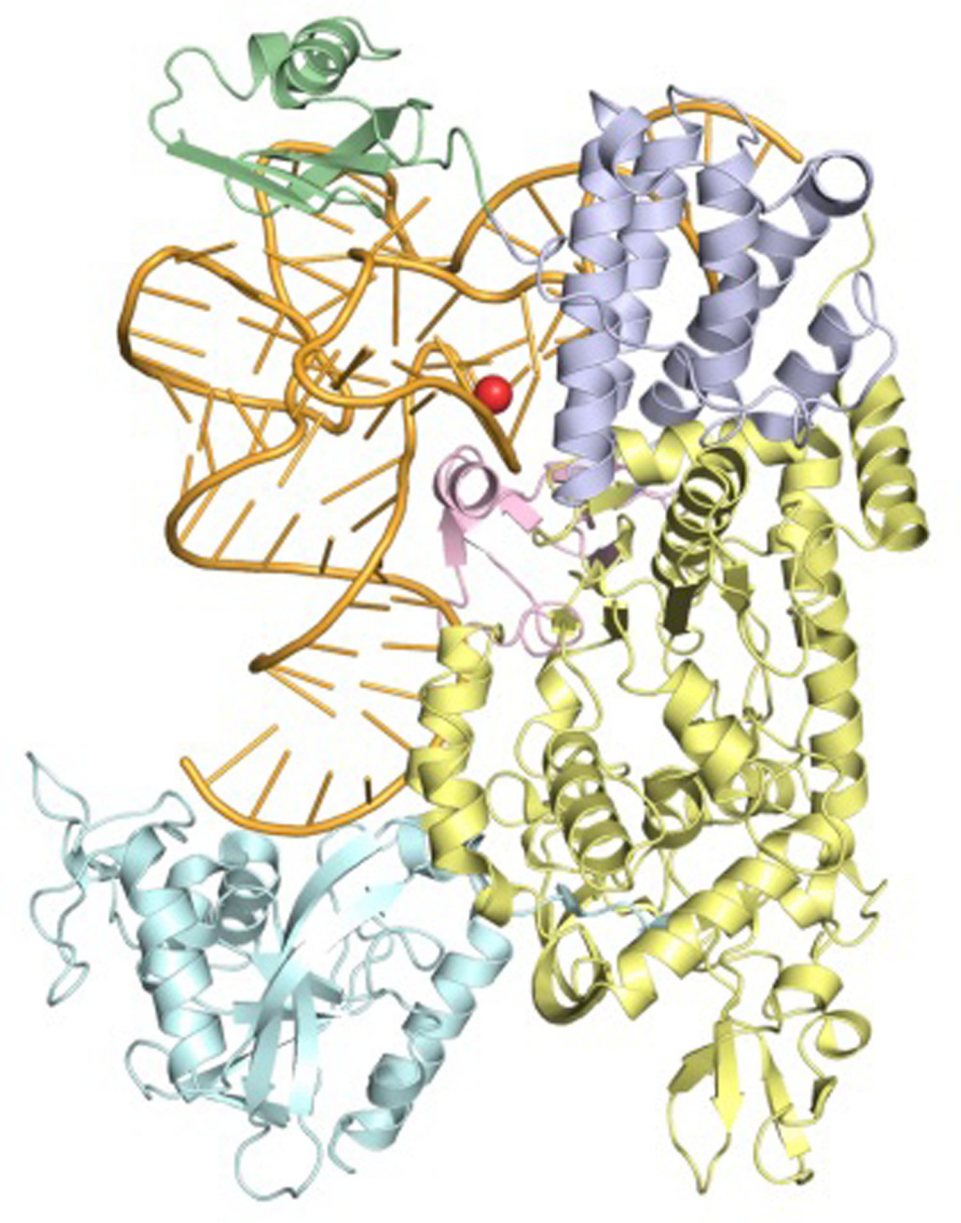
Ribbon representation of the LeuRS-tRNA^Leu^ complex from *Thermus thermophilus* determined by X-ray crystallography (PDB ID: 2BTE) at a Resolution of 2.9 Å. The structural domains of LeuRS are colored as follows: catalytic, yellow; leucine-specific, pink; editing, cyan; anticodon-binding, lilac; carboxy-terminal, green. The tRNA is colored orange and tRNA bases are shown as sticks. Position A14, equivalent to m.3243A, is highlighted by a red sphere.

Several 3D structures of LeuRS have been experimentally determined by X-ray crystallography and are available from the protein data bank (PDB; Berman et al., 2000). Although none of these structures are from mitochondria, sequence analyses reveals that both human and yeast mt LeuRS are closely related to cytoplasmic LeuRS from the bacteria *Thermus thermophilus* (Tukalo et al., 2005) and *Escherichia coli* (Palencia et al., 2012), whose 3D structures have been determined in complex with the cognate tRNA^Leu^ (**Figure 1**).

Analysis of the bacterial LeuRS structures (PDB IDs: 2BTE and 4AS1) revealed that LeuRS-Cterm interacts with the ‘elbow region’ of the cognate tRNA and establishes a higher number of contacts with the sugar-phosphate backbone than with nucleotide-specific chemical groups (13 and 3, respectively, in the higher resolution LeuRS-tRNA^Leu^ complex structure from *E. coli*). The preferred interaction of human mt-LeuRS-Cterm with ribose and phosphate oxygen atoms, which are present in all tRNAs, may contribute to explain its ability to bind to both cognate mt-tRNA^Leu(UUR)^ and non-cognate mt-tRNA^Ile^, and rescue defects associated with point mutations in both tRNAs. Additionally, analysis of the 3D models of human and yeast mt-LeuRS-Cterm, built by homology using the 3D structure of *E. coli* LeuRS as a template, showed that positive residues, which are relatively distant in the amino acid sequences are spatially clustered (Perli et al., 2014). This results in the formation of basic patches on the domain surfaces, which might explain the ability of both domains to be imported into mitochondria in spite of the lack of a canonical MTS.

## Future Perspectives

Currently, no reliable treatments or therapies are available for respiratory chain deficiencies due to mt-DNA encoded tRNA genes. Strategies as diverse as those aimed at mt tRNA delivery or mt ATP production increase have resulted in limited success.

Based on the evidences provided so far, the mt-LeuRS-Cterm is both an attractive new candidate for future therapeutic applications in mt-tRNA related diseases by itself, and opens a number of potential additional therapeutic avenues. In this regard it is worthwhile to identify: (i) smaller mt-LeuRS-Cterm fragments endowed with mt localization and rescuing ability; (ii) further mutations in mt-tRNAs aminoacylated by class I or II aaRS that can be rescued by mt-LeuRS Cterm and/or smaller peptides thereof; and (iii) additional aaRSs and/or peptides endowed with rescuing ability.

It has been recently demonstrated in the yeast model that the defective phenotype associated with human equivalent point mutations in *MTT* genes can be rescued by overexpressing short sequences (named β30_31 and β32_33, ∼15 amino acid long) derived from the human mt-LeuRS-Cterm (Francisci et al., 2011). This suggests that mt-LeuRS-Cterm-derived peptides may be used as therapeutic tools, provided that suitable agents for mitochondria targeting are developed to deliver them to their subcellular destination. Such small mt-LeuRS-Cterm peptides may even prompt the development of non-peptide organic molecules, especially if the rescuing activity can be further restricted to smaller regions.

Further studies on the yeast model have recently shown that overexpressed mt-LeuRS-Cterm and β30_31 and β32_33 peptides suppress the respiratory defects of the mutants in mt-tRNAs aminoacylated by class II, as well as class I, mt-aaRS (Di Micco et al., 2014).

Interestingly, the ability of the whole mt-LeuRS to rescue pathological mutations in both cognate tRNA^Leu(UUR)^ and non-cognate tRNA^Ile^ and mt-tRNA^Val^ has been shown to be shared by class Ia mt-IleRS and mt-ValRS in both human cells (Perli et al., 2014) and the yeast model (Montanari et al., 2010). However, at variance with mt-LeuRS-Cterm, the carboxy-terminal regions from mt-IleRS and mt-ValRS exerted very limited rescuing activities in the yeast model (Di Micco et al., 2014). Indeed, the absence of detectable sequence similarity between mt-LeuRS and mt-IleRS or mt-ValRS human or yeast and the different structure and tRNA binding mode detected in homologous enzymes of known 3D structure indicate that the properties of mt-LeuRS-Cterm are not necessarily shared by the carboxy-terminal domain of different aaRSs.

Finally, in view of potential therapeutic application, it is essential to further investigate the molecular mechanisms underlying the activity of mt-LeuRS-Cterm and its derived peptides, by measuring their effect on multiple parameters of mt function. It is expected that the recently reported availability of mouse models of mt-tRNA mutation related disease (Shimizu et al., 2014) will further boost research in this important medical field.
